# Malnutrition Is Associated with Protection from Rotavirus Diarrhea: Evidence from a Longitudinal Birth Cohort Study in Bangladesh

**DOI:** 10.1128/JCM.00916-16

**Published:** 2016-09-23

**Authors:** Hans Verkerke, Shihab Sobuz, Jennie Z. Ma, Sarah E. Petri, Dan Reichman, Firdausi Qadri, Mustafizur Rahman, Rashidul Haque, William A. Petri

**Affiliations:** a Division of Infectious Diseases and International Health, University of Virginia, Charlottesville, Virginia, USA; b International Centre for Diarrheal Disease Research, Bangladesh, Dhaka, Bangladesh; c Department of Public Health Sciences, University of Virginia, Charlottesville, Virginia, USA; Memorial Sloan-Kettering Cancer Center

## Abstract

Rotavirus is a leading cause of dehydrating diarrhea and death among infants and children globally, particularly in communities of the developing world. While numerous studies have described the complex relationships among infectious diarrhea, growth faltering, and poverty, the impact of nutritional status on susceptibility to rotavirus diarrhea is not well understood. In a longitudinal study conducted over the first 3 years of life among 626 slum-dwelling infants enrolled at birth in Dhaka, Bangladesh, we observed that common measures of healthy growth and development were positively associated with a risk of symptomatic rotavirus infection. This finding runs counter to the idea that improving childhood nutrition will implicitly decrease the incidence of symptomatic infection by enteric pathogens. As childhood nutrition improves worldwide, rotavirus infection may remain a public health challenge, making universal vaccination of even greater importance.

## INTRODUCTION

For more than a decade, we have studied the natural history of infants and children living in Mirpur, an urban slum of Dhaka, Bangladesh. These longitudinal studies of diarrhea and child development have uncovered significant environmental, genetic, and immunological correlates of diarrheal disease. They have also illustrated the devastating burden of enteric infections, which alter the absorption of critical nutrients, delay cognitive development, and interfere with oral vaccines (1–9).

Rotavirus is the most common cause of moderate to severe gastroenteritis among children under 5 years old globally, and dehydrating diarrhea associated with rotavirus is estimated to cause approximately 200,000 deaths annually (10). Many of these deaths occur in infants and children living in the developing world, where poor sanitary infrastructure facilitates fecal-oral transmission of numerous enteropathogens. In Bangladesh, approximately 6,000 deaths in children <5 years old are attributable to rotavirus diarrhea each year, amounting to an annual mortality rate of 38 per 100,000 children (11). Two licensed rotavirus vaccines, Rotarix (GlaxoSmithKline Biologicals, Belgium) and RotaTeq (Merck & Co., Inc., USA), have been widely applied in national immunization programs since 2006. Despite these widespread vaccination efforts, the Global Enteric Multicenter Study (GEMS) recently affirmed rotavirus as the leading cause of moderate to severe diarrhea among infants in all seven of its study sites spanning Africa and South Asia (12).

We undertook an analysis of rotavirus diarrhea in a birth cohort of 626 infants living in a slum of Mirpur Thana in Dhaka, the capital city of Bangladesh. These infants were not vaccinated against rotavirus and experienced a high prevalence of malnutrition at birth and throughout the first 3 years of life. In a univariable analysis of rotavirus diarrhea among infants who completed at least 3 years of observation, we observed an association between better nutritional status and a risk of experiencing symptomatic rotavirus infection in the first 3 years of life. Multiple hospital surveillance and cross-sectional studies have reported increased prevalence of rotavirus diarrhea among well-nourished compared to malnourished infants (13, 14). Notably, a hospital surveillance study conducted at the International Centre for Diarrheal Disease Research, Bangladesh, from 1993 to 2011, reported nearly double the incidence of rotavirus among overweight and obese compared to malnourished patients (14). Others have reported no correlation between nutritional status and incidence or an association between malnutrition and rotavirus diarrheal severity (13–20). While some of these observations provide evidence for a link between nutritional status and rotavirus diarrhea, others failed to find such an association, and all suffer from limitations inherent in hospital surveillance studies and thus may not be representative of epidemiological patterns in the community.

Based on our univariable analysis and data from published studies, we tested the hypothesis that nutritional status influences susceptibility to rotavirus infection in early life using a generalized mixed-effects model of anthropometric measures at 3-month intervals, with rotavirus diarrhea in each interval as the longitudinal outcome. To our knowledge, this is the first longitudinal report of a robust association between better nutritional status and susceptibility to rotavirus diarrhea.

## MATERIALS AND METHODS

### Study population and surveillance.

Pregnant mothers were recruited for this study from an urban slum of Mirpur Thana, Dhaka, Bangladesh. The study commenced in January 2008, and the last child completed follow-up in December 2012. Newborns were enrolled within 72 h of birth at home or in the study clinic. Subjects received free primary health care through the study community clinic throughout the duration of the study. Written informed consent was obtained for each infant from a parent or legal guardian. Prior to enrollment, approval for this study was obtained from the institutional review board of the University of Virginia and the ethics board of the International Center for Diarrheal Disease Research, Bangladesh (ICDDR,B).

Socioeconomic data, including monthly income and years of formal maternal education, were assessed by a survey at the time of enrollment. Exclusive and nonexclusive breastfeeding practices were monitored by self-reporting and field observation throughout infancy. Field research assistants (FRAs) made home visits every other day to track the medical history of each infant, focusing on diarrheal illness. Parents of sick infants were encouraged to visit the study clinic for assessment and treatment by a study medical officer. FRAs also made referrals to the field clinic or hospital in cases of acute illness evident at the time of home visits.

Mothers or family members reported diarrheal episodes, defined as ≥3 unformed bowel movements in a 24-h period, or by the mother's definition for a breastfed child to FRAs, who collected stool samples. By definition, episodes of diarrhea required separation by at least 3 days without diarrhea to be considered independent episodes. Within 3 h of collection, all stool collected from diarrheal episodes was transported on ice from the field clinic to the parasitology laboratory at the ICDDR,B. Diarrheal stool was then aliquoted before storage at −80°C.

### Detection of rotavirus.

Group A rotavirus was detected in fresh or frozen diarrheal stool samples using the ProSpecT Rotavirus enzyme-linked immunosorbent assay (ELISA) (catalog no. R240396). All samples collected from diarrheal episodes in the study window were tested by ELISA for rotavirus antigen.

G and P genotyping was carried out on 200 out of the 227 rotavirus-positive diarrheal stool samples using a genotype-specific multiplex reverse transcriptase PCR. This assay was capable of detecting four G genotypes (G1, G2, G9, and G12) and three P genotypes (P[4], P[8], and P[6]), as previously described (21). Genotyping was performed on samples collected between 2008 and 2011 and was not extended to samples collected in 2012, because a full analysis of the genotypes in every sample collected was beyond the scope of this study.

### Anthropometry.

Maternal height and weight were assessed, and body mass index was calculated by dividing the mass of the mother in kilograms by her height in meters squared. Infant anthropometric measures were collected by FRAs at birth and at 3-month intervals. Height-for-age Z-scores (HAZ), weight-for-age Z-scores (WAZ), and weight-for-height Z-scores (WHZ) were calculated using the WHO Anthro software, version 3.0.1, which compared study subjects to age- and sex-matched members of the World Health Organization (WHO) reference population (22).

### Statistical analyses.

Demographic, socioeconomic, anthropometric, and medical history data were entered into the study database. Median values with interquartile ranges for skewed continuous variables are reported. Nonparametric Mann-Whitney tests were used to compare central trends of continuous variables, and chi-square and Fisher's exact tests were used where appropriate to compare categorical variables.

All analyses were performed using SAS 9.3 (SAS Institute, Inc., Cary, NC, USA). Longitudinal analyses were conducted to test the hypothesis that nutritional status was associated with risk of rotavirus diarrhea in the first 3 years. In a generalized mixed-effects model, the presence or absence of rotavirus-positive diarrhea over each 3-month interval from birth to the end of 3 years was considered the longitudinal response. Anthropometric measures at the beginning of each 3-month interval were the time-varying predictors in our models. A separate analysis was performed for each anthropometric measure, with adjustment for fixed factors, including gender, maternal age, maternal body mass index (BMI), maternal education, family income, and a time-varying categorical indicator of exclusivity of breastfeeding during each interval. The Proc GLIMMIX in SAS was used for the generalized mixed-effects analyses. Additionally, the effect of first rotavirus infection on the subsequent rotavirus infection was evaluated in Cox regression with Proc PHREG in SAS, where repeated rotavirus infections were considered recurrent events. The recurrent event regression analysis was adjusted for the same set of risk factors as in the mixed-effects model. *P* values of <0.05 were considered statistically significant.

## RESULTS

### Study population and anthropometry.

Six hundred twenty-six infants born in an urban slum of Mirpur, a neighborhood of Dhaka, Bangladesh, were enrolled at birth between January 2008 and December 2012. Of the infants, 80.5% (*n* = 504) completed at least 1 year of observation, 71.0% (*n* = 445) completed at least 2 years of observation, and 47.6% (*n* = 298) completed at least 3 years of observation. Of the infants, 52.4% (*n* = 328) had dropped out of the study before completing 3 years of observation, our inclusion criterion for the univariable analysis conducted. Infants who dropped out during the study window did not differ significantly in maternal education or age, family size, monthly income, or baseline anthropometric traits from the total group enrolled (data not shown).

**FIG 1:**
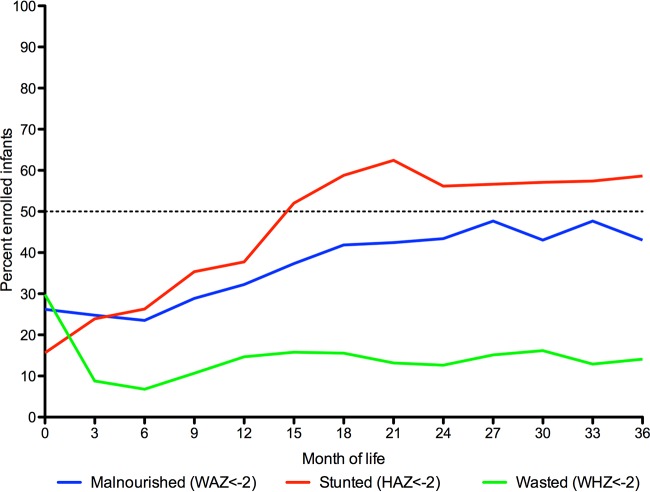
Longitudinal monitoring of infant anthropometry in the first 3 years of life. To estimate the trajectory of changing nutritional status, infant anthropometric measures were tracked at 3-month intervals in the first 3 years of life. The percentages of the population that were malnourished (WAZ, <−2), stunted (HAZ, <−2), and wasted (WHZ, <−2) are plotted at each 3-month time point from birth to age three.

Anthropometric trends in the first 3 years of life for the study population are traced in Fig. 1. Low weight-for-age (WAZ, <−2), stunting (HAZ, <−2), and wasting (WHZ, <−2) were relatively common in this population at birth at 26%, 16%, and 30% incidence, respectively. The proportion of the population that was underweight or stunted rose over the first 3 years of life to more than half. The proportion of the population that was wasted fell in the first 3 months and remained close to 10% throughout the remaining months of the first 3 years of life.

### Incidence of diarrhea and rotavirus diarrhea.

Diarrheal episodes were reported by mothers or family members to field research assistants (FRAs) during home visits, or during sick visits to the study clinic. Stool samples were collected in the home or at the field clinic to identify potentially causal enteropathogens. Overall, 4,487 diarrheal episodes were reported in infants zero to 3 years of age from January 2008 to December 2012. Of this subset of reported episodes, 61.5% (*n* = 2,758) of stool samples were collected and transported to the ICDDR,B for testing. The total number of diarrheal episodes reported declined as infants aged, from 339 episodes per 100 infants from birth to age one to 171 episodes per 100 children in the third year of life.

Overall, 227 episodes of rotavirus-positive diarrhea were reported and collected from 190 children during the study window. Restricting our analysis to the first 3 years of life, 188 infants experienced 223 rotavirus-positive diarrheal episodes. Pathogen diversity was high, with 36.3% of rotavirus-positive diarrheal stools coinfected with Entamoeba histolytica, Giardia lamblia, or Cryptosporidium parvum. The majority of infants in the population affected by rotavirus diarrhea, 83.5% (*n* = 157), experienced only one rotavirus-positive diarrheal episode. Of the affected infants, 14.4% (*n* = 27) experienced two rotavirus-positive episodes. Only 2.1% (*n* = 4) of the affected infants experienced three rotavirus-positive episodes, and no infants experienced greater than three rotavirus-positive episodes in the study window.

**FIG 2:**
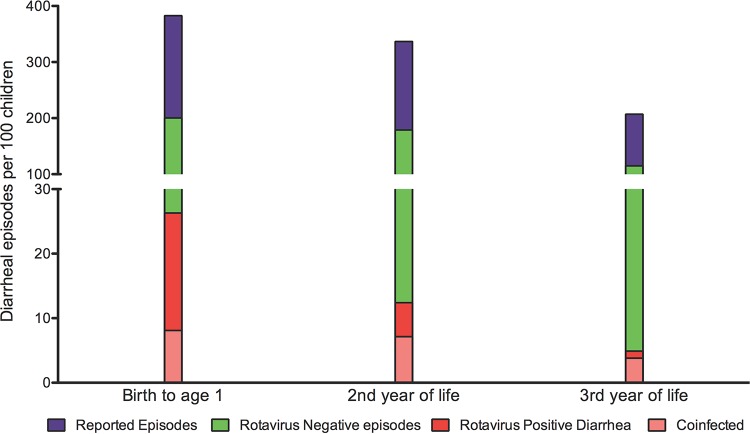
Experience with diarrhea and rotavirus-positive diarrhea in the first 3 years of life. Diarrheal surveillance data were available for 626 infants in the first year of life, 504 infants in the second year of life, and 445 infants in the third year of life. Each bar represents the total number of reported episodes per 100 infants in a given period of life. Three hundred eighty-three episodes of all-cause diarrhea were reported per 100 infants in the first year of life, 336 in the second year of life, and 206 in the third year of life. Each bar is further subdivided into the number of rotavirus-negative (green) and rotavirus-positive (red) diarrheal episodes per 100 children in a given age group. The number of coinfections with rotavirus and Entamoeba histolytica, Giardia lamblia, or Cryptosporidium parvum per 100 children is shown in pale red.

The number of infants who experienced rotavirus-positive diarrhea declined in the second and third years of life. Overall, 35.6 rotavirus-positive diarrheal episodes were detected per 100 infants in their first 3 years of life. The majority (23.3 per 100 infants) were detected in the first year of life (Fig. 2).

### Clinical characteristics of rotavirus-positive diarrheal episodes.

Rotavirus-positive diarrhea was more common among younger infants (*P* < 0.0001) and was associated with significantly higher diarrheal severity by Vesikari score (*P* < 0.0001). Fever (*P* = 0.0001) and vomiting (*P* < 0.0001) were also more commonly associated with rotavirus-positive diarrheal episodes than with rotavirus-negative episodes. Included in our univariable analysis were all diarrheal episodes and rotavirus-positive diarrheal episodes collected during the study window from January 2008 to December 2012, without regard to child age at the time of collection (Table 1).

**TABLE 1:** Clinical characteristics of collected rotavirus-positive and rotavirus-negative diarrheal episodes^*a*^

Parameter	Total	Rotavirus-positive episodes	Rotavirus-negative episodes	*P* value
No. of patients	2,758	227	2,531	
Age (mo)	13.5 (6.6–22.6)	9.4 (5.9–15.3)	14.2 (6.6–23.0)	<0.0001^*b*^
Duration (days)	1 (1–1)	1 (1–1)	1 (1–1)	0.6^*b*^
Severity (Vesikari score [IQR])	4 (4–6)	5 (4–7)	4 (4–5)	<0.0001^*b*^
% affected by:				
Vomiting	5.6	17.6	4.5	<0.0001^*c*^
Fever	6.4	12.3	5.8	0.0001^*c*^

a All data are presented as the median and interquartile range (IQR), unless otherwise stated.

b Mann-Whitney *P value*.

c χ^2^ *P* value.

### Evidence for naturally acquired immunity against rotavirus infection conferred by symptomatic rotavirus diarrhea.

**FIG 3:**
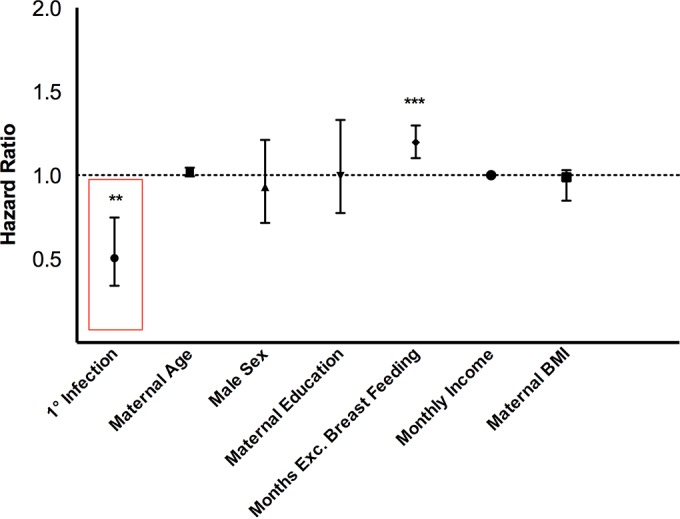
Primary rotavirus infection decreases the risk of subsequent rotavirus infection. We used a Cox regression to estimate the independent time-dependent impact of a primary symptomatic rotavirus infection on the risk of secondary symptomatic infection among infants in our cohort in the first 3 years of life. Maternal age at birth, male sex, maternal education, cumulative months of exclusive (Exc.) breastfeeding, monthly income, and maternal BMI at birth were included in the model as time-independent covariates. Hazard ratio estimates are plotted with 95% confidence intervals for each variable included in the survival model. * indicates the level of significance, with ** indicating a *P* value of <0.001 and ** a *P* value of <0.0001.

**FIG 4:**
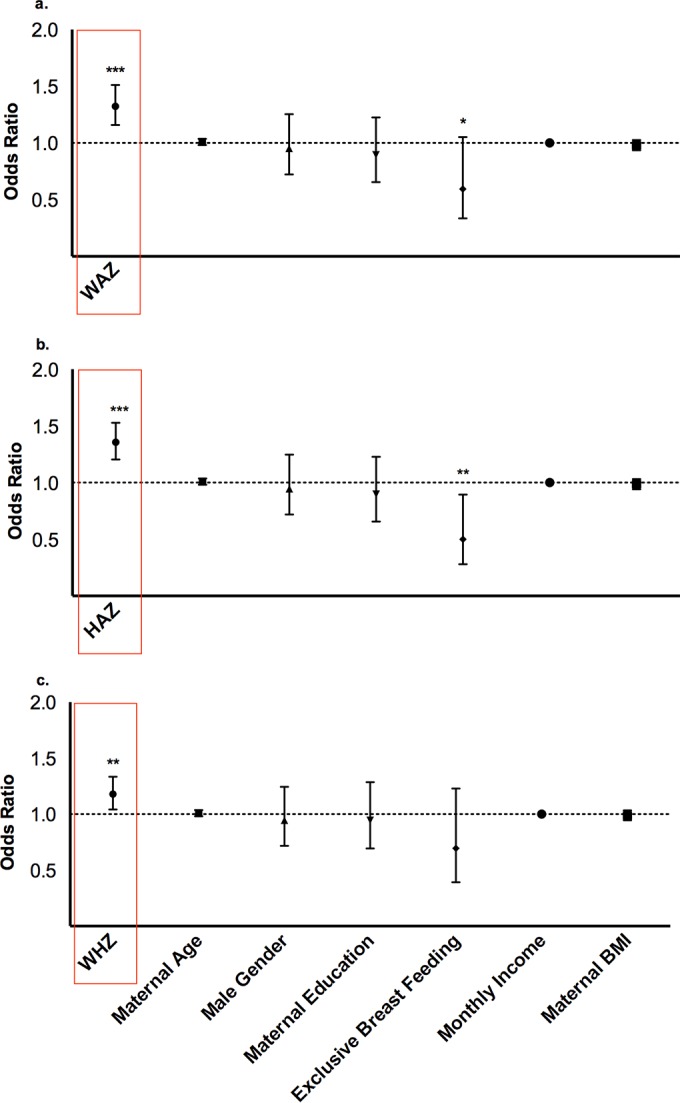
Malnutrition, stunting, and wasting reduce the risk of rotavirus infection in the first 3 years of life. Odds ratios for rotavirus diarrhea from longitudinal models of anthropometric Z-scores, boxed in red, are plotted with 95% confidence intervals. Weight-for-age (a), height-for-age (b), and weight-for-height Z-scores (c) were measured at 3-month intervals. Models for each anthropometric measure were adjusted for maternal age, infant sex, maternal education, monthly income, and maternal BMI. Exclusivity of breastfeeding during each interval was included as a time-varying covariate in each model. * indicates the level of significance, with ** indicating a *P* value of <0.001 and ** a *P* value of <0.0001.

The first rotavirus-positive episode of diarrhea was associated with protection of infants from subsequent symptomatic infection. Using a Cox regression analysis adjusted for sex, maternal age and body mass index (BMI) at birth, monthly income, maternal education, and duration of exclusive breastfeeding, we observed a protective effect of primary rotavirus diarrhea against subsequent symptomatic infection of between 34.0% and 74.7% (*P* = 0.0006). Overall duration of exclusive breastfeeding was also significantly associated with repeated symptomatic infection (*P* < 0.0001) (Fig. 3). While this result is somewhat surprising, we suspect the correlation to be unrelated to the overall protective effect of exclusive breastfeeding against infections early in life. In fact, we observed the opposite trend, shown in Fig. 4, with exclusivity of breastfeeding protecting infants over 3-month durations.

### Seasonality and genotype distribution of rotavirus-positive diarrheal episodes.

Three seasonal peaks in rotavirus diarrhea were observed in the six distinct seasons of the Bengal Delta region. Of the rotavirus-positive diarrhea, 22.9%, 20.6%, and 24.2% occurred in the monsoon (Borsha), late autumn (Hemonto), and spring (Bosonto) seasons, respectively. Fewer episodes were observed during the summer (Grish), autumn (Shorot), and winter (Shít) seasons (data not shown).

The subset of 200 rotavirus-positive diarrheal stool samples that were collected between 2008 and 2011 were genotyped for common VP7 (G) and VP4 (P) rotavirus variants. The major G genotypes observed in diarrheal stool between 2008 and 2011 were G12 (20%), G2 (20%), and G9 (38%). The major P genotypes observed during the same interval were P[8] (47%) and P[4] (29%). The major combined genotypes reflected the overall prevalence of G and P variants in the sampled population. G9P[8] accounted for 28%, G2P[4] for 19.5%, and G12P[6] for 9.5% of the samples tested. Eight percent of the samples could not be typed by the method used, and 6% were detectable mixed-genotype infections (data not shown).

### Univariable analysis of rotavirus diarrhea in the first 3 years of life.

To determine correlates of rotavirus diarrhea in the first 3 years of life, data were censored to include only the 298 infants who completed at least 3 years of observation. This subset did not differ significantly in baseline and demographic characteristics from the total enrollment or the infants who dropped out of the study (data not shown).

Out of these 298 infants, 38.6% (*n* = 115) experienced at least one episode of rotavirus-positive diarrhea in the first 3 years of life, and 61.4% (*n* = 183) did not experience rotavirus-positive diarrhea. Children who were not stunted at birth (HAZ, ≥−2) were more likely to be affected by rotavirus-positive diarrhea in the first 3 years of life (*P* = 0.01). Gender, overall duration of exclusive breastfeeding, maternal education or age, family size, income, and maternal BMI were not significantly different between the affected and unaffected groups in this analysis (Table 2).

**TABLE 2:** Comparison of infants affected and unaffected by rotavirus diarrhea in first 3 years of life^*a*^

Parameter^*b*^	Total	Rotavirus-affected infants	Rotavirus-unaffected infants	*P* value
No. of patients	298	115	183	
Maternal and family characteristics				
Maternal education (yr)	4 (0–7)	3 (3–12)	4 (4–12)	0.44^*c*^
Family size	5 (4–7)	5 (4–6)	5 (4–7)	0.68^*c*^
Mo income (BDT)	6,000 (4,400–8,500)	6,200 (4,500–8,000)	6,000 (4,250–8,700)	0.85^*c*^
Maternal age (yr)	25 (22–29)	25 (22–30)	25 (21–28)	0.06^*c*^
Maternal BMI	20.7 (19.0–37.5)	20.8 (19.2–23.3)	20.7 (18.8–22.8)	0.35^*c*^
Anthropometry and breastfeeding				
% exclusive breastfeeding >6 mo	49.7	29.6	18.6	0.13^*d*^
Birth Z-scores				
% stunted (HAZ<−2)	15.4	8.7	19.7	0.01^*d*^
% undernourished (WAZ<−2)	25.5	22.6	27.3	0.41^*d*^
% wasted (WHZ<−2)	27.9	27.8	27.9	1.00^*d*^
% male	53.0	53.9	52.4	0.81^*d*^

a All data are presented as the median and interquartile range (IQR), unless otherwise stated.

b BDT, Bangladeshi Taka; HAZ, height-for-age Z-score; WAZ, weight-for-age Z-score; WHZ, weight-for-height Z-score.

c Mann-Whitney *P* value.

d Fisher's exact *P* value.

### Nutritional status is positively associated with rotavirus diarrhea in the first 3 years of life.

Using a longitudinal mixed-effects model of rotavirus diarrhea and changing anthropometric measures, we observed a significant association between better nutritional status and rotavirus infection during each 3-month interval of infancy. For each one-unit increment in HAZ, there was a 36.0% higher risk of rotavirus diarrhea (odds ratio [OR], 1.36; 95% confidence interval [CI], 1.20 to 1.53; *P* < 0.0001). This observation also held true for the two other anthropometric measures (WHZ and WAZ), where better-nourished and nonwasted infants were more susceptible to rotavirus diarrhea. Specifically, for each one-unit increment in WAZ and WHZ, the risk of rotavirus diarrhea increased by 32% and 18%, respectively (OR for WAZ, 1.32; 95% CI, 1.16 to 1.51; *P* < 0.0001; and OR for WHZ, 1.18; 95% CI, 1.04 to 1.33; *P* = 0.009). In Fig. 4a to c, the odds ratios are reported from our mixed-effects modeling of anthropometric measures as predictors of rotavirus diarrhea over 3-month intervals. We also saw a trend toward decreased risk of rotavirus diarrhea associated with the self-reported practice of exclusive breastfeeding over each interval.

## DISCUSSION

The most important finding we report is that better nutritional status among Bangladeshi infants was strongly associated with a higher risk of rotavirus diarrhea in the first 3 years of life. This association was consistent for three anthropometric measures: malnutrition, stunting, and wasting. Our findings may have important implications as populations in the developing world, chronically exposed to rotavirus, attain increasing levels of food security. Our results suggest that rotavirus diarrhea will remain a recalcitrant public health problem despite or even because of projected gains in infant nutritional status in low-income countries. This possibility serves to highlight the vital importance of global rotavirus vaccination efforts. One limitation of our study is that not all reported diarrheal episodes could be collected. There is a possibility, therefore, that the true incidence of rotavirus diarrhea is higher in the community than we report. While we acknowledge this limitation, we consider the conclusion, that malnutrition is associated with a decreased incidence of symptomatic rotavirus infection, to be a valid one, since we expect underreporting to affect the measured incidence of diarrhea similarly in well-nourished and malnourished infants.

Malnutrition affects the physiological integrity of the gut, the potency and specificity of the enteric immune system, susceptibility to enteric pathogens (23), and the composition of the gut microbiota (24, 25). It is thus not unreasonable to postulate that differences along one or more of these dimensions associated with malnutrition contribute to rotavirus susceptibility in early life. Malnourished infants living in communities with poor sanitation are at heightened risk of developing environmental enteropathy (EE), a pathology of the gut associated with chronic intestinal inflammation and shortening of the enterocytic villi (26). It is possible that the shortening of villi in malnourished infants may inhibit or altogether abrogate rotavirus entry and replication. Interestingly, oral polio vaccines and oral rotavirus vaccines, which rely on live attenuated virus for immunization, exhibit reduced efficacy in the developing world (27). This reduced efficacy, at least for the oral polio vaccine, may in part be due to reduced vaccine uptake among malnourished infants with EE. If malnutrition-associated EE is indeed a mechanism of live attenuated oral vaccine failure, it is reasonable to hypothesize that rotavirus and perhaps other enteric viruses are better able to replicate in the healthy enteric niche of a well-nourished infant without EE.

Malnutrition is also associated with dramatic and persistent changes in gut microbial ecology. These changes alter enteric immunity, absorption, and metabolite composition at the interface of enteric infection (24, 25, 28–30, 33). Recently, Jones et al. developed the first system for *in vitro* culture of norovirus, another important enteropathogen. They found that coculture with certain gut bacterial species dramatically improved viral entry and survival in culture (31). It is likely that similar metabolic cross talk is crucial for infectivity of other enteric viruses and may account for the persistent challenge of culturing rotavirus from primary clinical isolates.

While these mechanisms are intriguing and plausible, it is impossible in the present study to do more than speculate about the cause of the strong association we observed. The challenge of measuring the many sequelae attributable to malnutrition and stunting is persistent in the field of global health. Noninvasive measures of EE, for instance, are critically needed. As technologies and models of disease and growth improve, our ability to attribute risk and outcome will improve. The analysis we present here provides important, but limited, insights into the role of malnutrition in rotavirus disease. More studies are needed to determine cause. Longitudinal and case-control studies in diverse human populations are needed to confirm that this association is broadly relevant. Animal and *in vitro* models of rotavirus infection will also continue to yield critical insights into the interactions among nutrition, immunity, microbial ecology, and enteric viruses. However, the positive association we observe between nutritional status and rotavirus diarrhea susceptibility highlights the importance of combining global vaccination efforts with nutritional interventions and programs to reduce transmission. Nutritional interventions alone may fail to reduce rotavirus diarrhea effectively. Encouragingly, the WHO Expanded Programme on Immunization (EPI) has added rotavirus vaccines to the growing list of globally accessible immunizations. Mortality associated with diarrhea dropped by 41% in Mexico following the introduction of rotavirus vaccines, demonstrating the impressive efficacy of such programs (32).
